# Mild hypothermia provides Treg stability

**DOI:** 10.1038/s41598-017-10151-1

**Published:** 2017-09-20

**Authors:** Natalia Marek-Trzonkowska, Karolina Piekarska, Natalia Filipowicz, Arkadiusz Piotrowski, Magdalena Gucwa, Katrin Vogt, Birgit Sawitzki, Janusz Siebert, Piotr Trzonkowski

**Affiliations:** 1 Laboratory of Immunoregulation and Cellular Therapies, Department of Family Medicine, Medical University of Gdańsk, ul. Dębinki 2, 80-210 Gdańsk, Poland; 2 Department of Biology and Pharmaceutical Botany, Medical University of Gdańsk, al. Gen. J. Hallera 107, 80-416 Gdańsk, Poland; 3 Institute for Medical Immunology, Charité – Universitätsmedizin Berlin, corporate member of Freie Universität Berlin, Humboldt-Universität zu Berlin and Berlin Institute of Health, Augustenburgerplatz 1, 13353 Berlin, Germany; 4 Department of Family Medicine, Medical University of Gdańsk, ul. Dębinki 2, 80-210 Gdańsk, Poland; 5 Department of Clinical Immunology and Transplantology, Medical University of Gdańsk, ul. Dębinki 7, 80-210 Gdańsk, Poland

## Abstract

Regulatory T cells (Tregs) play crucial role in maintenance of peripheral tolerance. Recent clinical trials confirmed safety and efficacy of Treg treatment of deleterious immune responses. However, Tregs lose their characteristic phenotype and suppressive potential during expansion *ex vivo*. Therefore, multiple research teams have been studding Treg biology in aim to improve their stability *in vitro*. In the current paper, we demonstrate that mild h*y*pothermia of 33 °C induces robust proliferation of Tregs, preserves expression of FoxP3, CD25 and Helios, and prevents TSDR methylation during culture *in vitro*. Tregs expanded at 33 °C have stronger immunosuppressive potential and remarkably anti-inflammatory phenotype demonstrated by the whole transcriptome sequencing. These observations shed new light on impact of temperature on regulation of immune response. We show that just a simple change in temperature can preserve Treg stability, function and accelerate their proliferation, responding to unanswered question- how to preserve Treg stability *in vitro*.

## Introduction

Thymus derived, natural regulatory T cells (Tregs) constitute ≈1% of lymphocytes in peripheral blood, but they play a crucial role in maintenance of peripheral tolerance^1,2^. Lack of functional Tregs leads to onset of multiple autoimmune diseases and hypersensitivity as it is observed in immune dysregulation, polyendocrinopathy, entheropathy, X-linked syndrome (IPEX)^3^. Tregs can be called “intelligent steroids” as physiologically their immunosuppressive activity is targeted against pathological responses (e.g. autoimmunity, excessive inflammation, hypersensitivity) and do not impair immunity to infectious agents. Results of clinical trials, including our studies, confirmed that Tregs are safe therapeutic tool for treatment of deleterious immune responses^4–10^.

Our team has been working on biology and clinical application of Tregs for more than 10 years. We were the first group who administered *ex vivo* expanded Tregs in human in graft versus host disease (GVHD)^4^, in type 1 diabetes (DM1)^5–7^ and recently in multiple sclerosis^11^. Currently, multiple different research teams have been tested *ex vivo* expanded Tregs in prevention and therapy of GVHD, DM1, and tolerance induction in kidney and liver transplantation (numbers of clinical trials listed in the ref.^11^). Thus, during the last 5 years a dynamic sprout of Treg based therapies has been observed.

In general, all Treg trials have the common goal- to provide an intelligent therapy that will inhibit deleterious immune reactions with no impact on physiological immunity. However, at the same time research groups conducting these studies struggle with the same technical problems that are: variable Treg proliferation rate and continuous loss of their characteristic phenotype and function during culture *in vitro* ^9,12–14^.

For clinical purposes Tregs are used to be expanded for up to 14 days in presence of IL-2 and magnetic beads coated with anti-CD3 and anti-CD28 antibodies (Ab)^5–7,9,15^. However, even in these established culture conditions and in the same laboratory, the fold increase of Treg number may vary between the expansions from several to nearly 50 times^9,15^. Hippen *et al*. reported in 3 experiments ≈3000 and ≈50 million-fold expansion of Tregs after 14 and 55 days, respectively. Nevertheless, instead of beads they restimulated Tregs with anti-CD3 Ab loaded KT64/86 cell line^16^. KT64/86 cells were recently used for clinical Treg expansion^10^, however the risk of cancer cell implementation to the clinical protocol of Treg culture has to be always considered.

An important and unsolved problem related to Treg expansion *in vitro* is a continuous decrease in frequency of FoxP3^+^ cells^12,14^. Even highly purified by fluorescence-activated cell-sorting (FACS) CD4^+^CD25^High^CD127^-/Low^ T cells (≈100% post-sort purity) present variable proportions of FoxP3^+^ cells (50–75%) after 2–3 weeks of expansion *in vitro* ^12,14^. As FoxP3 downregulation results in loss of regulatory function of Tregs^17^, this is a serious problem for all Treg based clinical therapies. At some point rapamycin seemed to be the solution for this issue. However, recent studies confirm that the drug preferentially inhibits proliferation of conventional T cells that can contaminate Treg culture^18^, but does not prevent loss of FoxP3 expression in natural Tregs during the second week of expansion^13,19^. Noteworthy, 5-10-fold reduction in Treg proliferation in presence of rapamycin was also reported^16^.

It has been demonstrated that stable FoxP3 expression in natural Tregs depends on selective demethylation of Treg-specific demethylated region (TSDR) of FoxP3 gene^20^. Nevertheless, it was also reported that during expansion *in vitro* TSDRs undergo methylation even in natural Tregs. This phenomenon was accompanied by continuous decrease in FoxP3 expression, and suppressive activity of Tregs, as well as increasing production of proinflammatory cytokines^12^. Thus, in the context of clinical trials, an improvement of current Treg expansion protocols is required to prevent TSDR methylation, loss of FoxP3 expression and Treg function *in vitro*.

In the present paper, we describe how to preserve Treg phenotype and function during culture *in vitro* without using chemical compounds affecting cell viability and safety of potential clinical therapy. We present how to efficiently expand stable and highly suppressive FoxP3^High^ Tregs with unmethylated TSDRs by changing only a temperature of cell culture. Presented protocol makes the final result of Treg expansion more predictable, than it was reported before. Pure and stable Treg population after expansion can help to standardize Treg therapy, and thus make it safer and more efficient. The strategy can be also used for effective expansion of rare antigen-specific Treg clones that seem to be the most promising tool for therapy of autoimmune diseases. Finally, presented data shed new light on temperature- thought to be the fixed and not manipulable parameter in Treg culture, as well as help to better understand regulation of Treg responses.

## Results

### Temperature of 33 °C induces robust proliferation of Tregs

**Figure 1:**
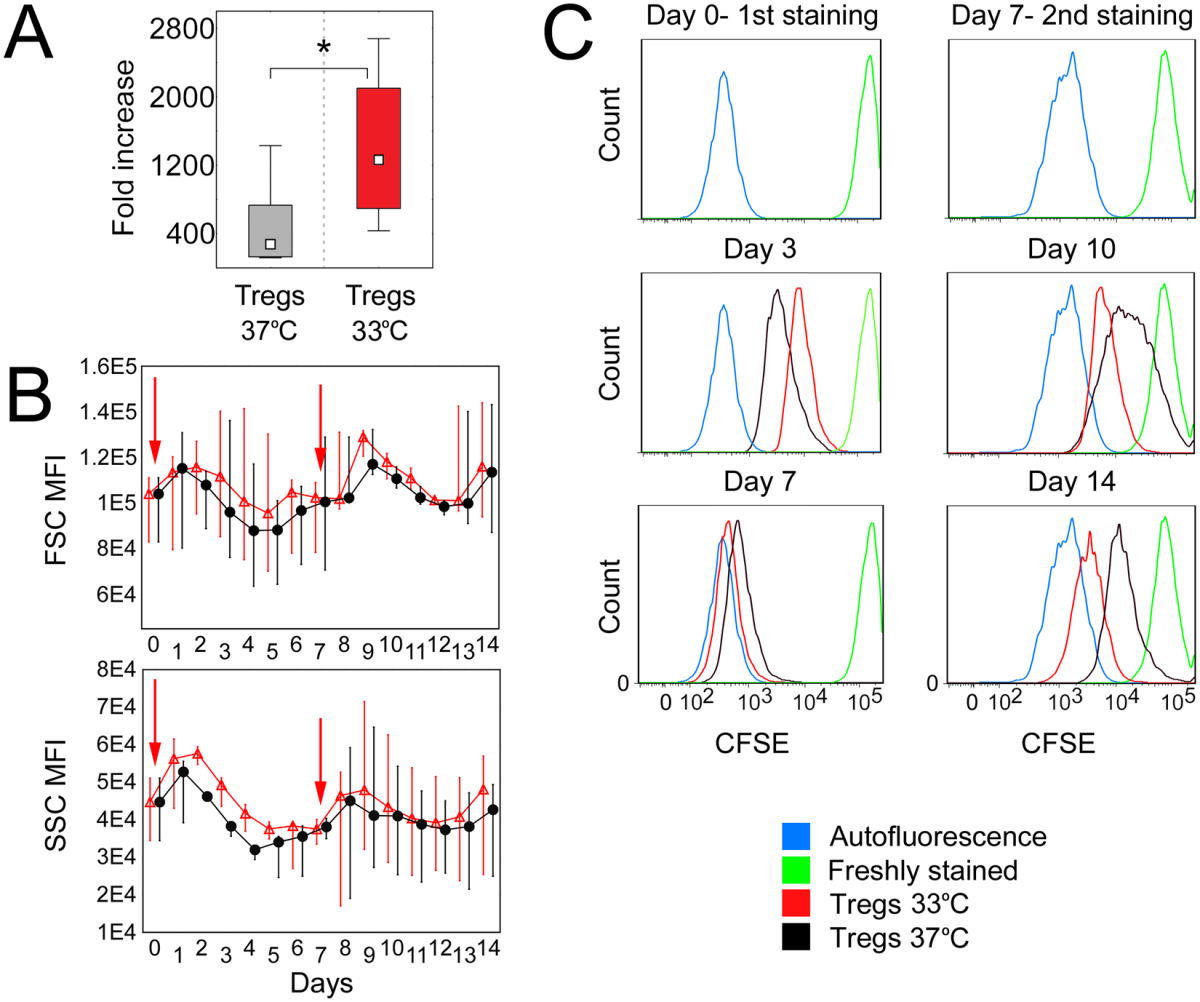
Mild hypothermia of 33 °C stimulates Treg proliferation. (**A**) Fold increase of initial Treg number after 14-day expansion at 37 and 33 °C (gray and red symbols, respectively; n = 11). Statistical differences were calculated with Mann- Whitney U test and are presented as medians (min.-max.). *p < 0.05. (**B**) 14-day follow-up of size (FSC) and granularity (SSC) of Tregs cultured at 37 (black symbols) and 33 °C (red symbols; n = 4). Arrows point the days of cell stimulation with anti-CD3/CD28 beads. Med﻿ian fluorescence intensity (MFI) of FSC and SSC ﻿is presented as value to power of 4 or 5 (e.﻿g. 8E4 = 8 × 10^4^).(**C**) 14-day follow-up of proliferation dynamics of CFSE stained Tregs cultured at 37 (black histograms) and 33 °C (red  histograms). Results of 1 of 4 representative experiments are shown for day 0 (1^st^ CFSE staining), day 3, day 7 before and after 2^nd^ CFSE staining, day 10 and day 14. Blue and green histograms depict fluorescence of unstained and freshly stained cells, respectively.

14-day culture of Tregs at 33 °C resulted in 4.5-fold higher cell counts as compared with Tregs expanded at 37 °C (median fold increase 275.52 vs 1261.5, respectively, Mann–Whitney U test, MW, p = 1 × 10^−3^; Fig. 1A). Daily analysis of size and granularity of Tregs expanded at 37 and 33 °C showed that cells cultured at mild hypothermia were slightly bigger and more granular at each time-point, than Tregs expanded at standard culture temperature. However, the differences were not statistically significant (Fig. 1B). In addition, different proliferation dynamics for Tregs expanded at 33 and 37 °C were observed. During the first 4 days of the culture Tregs kept at 33 °C revealed lower proliferation rate than those at 37 °C. Nevertheless, after this period a significant acceleration in their proliferation has been observed and since day 7 Treg counts at 33 °C were higher, than at 37 °C. Intensive Treg proliferation at 33 °C was kept until the end of 14-day cultures, while an inverse trend was observed at 37 °C (Fig. 1C).

In 2/13 cultures at 37 °C Tregs proliferated significantly less extensively (fold increase 16.26 and 32), than it was observed at this temperature typically. No similar phenomenon was observed when cells from the same donors were expanded at 33 °C (fold increase 1446.1 and 1130, respectively). However, because of significant deviation, these 2 experiments were not included into statistical calculations.

Further reduction of culture temperature to 29 °C almost completely ceased Treg proliferation (average fold increase <3). While hyperthermia of 39 °C has less deleterious impact on Tregs and the cells passed significantly more rounds of divisions at these conditions than at 29 °C. However, fold expansion at 39 °C was significantly lower than at 37 and 33 °C (127.1 vs 730 and 1261.5, respectively).

All Treg cultures were characterized by high post-sort purity and no significant deviation in this parameter was observed between the samples. On average after the isolation 98.9% (median, range 97–99.5%) of the cells presented CD3^+^CD4^+^CD25^High^CD127^−/Low^lin^−^doublet^−^ Treg phenotype and median contamination with CD4^+^CD25^−^, CD4^+^CD127^High^ and CD4^+^CD25^−^CD127^High^ cells, all considered as CD4^+^ effector T cells (Teffs), was equal to 0.4%, while non-Th (CD4^−^) cells constituted 0.6% (median) of the sorted population.

### Temperature of 33 °C enhances expression of FoxP3, CD25, CTLA-4, CD39 and Helios in Tregs and keeps Treg phenotype stable during culture *in vitro*

**Figure 2:**
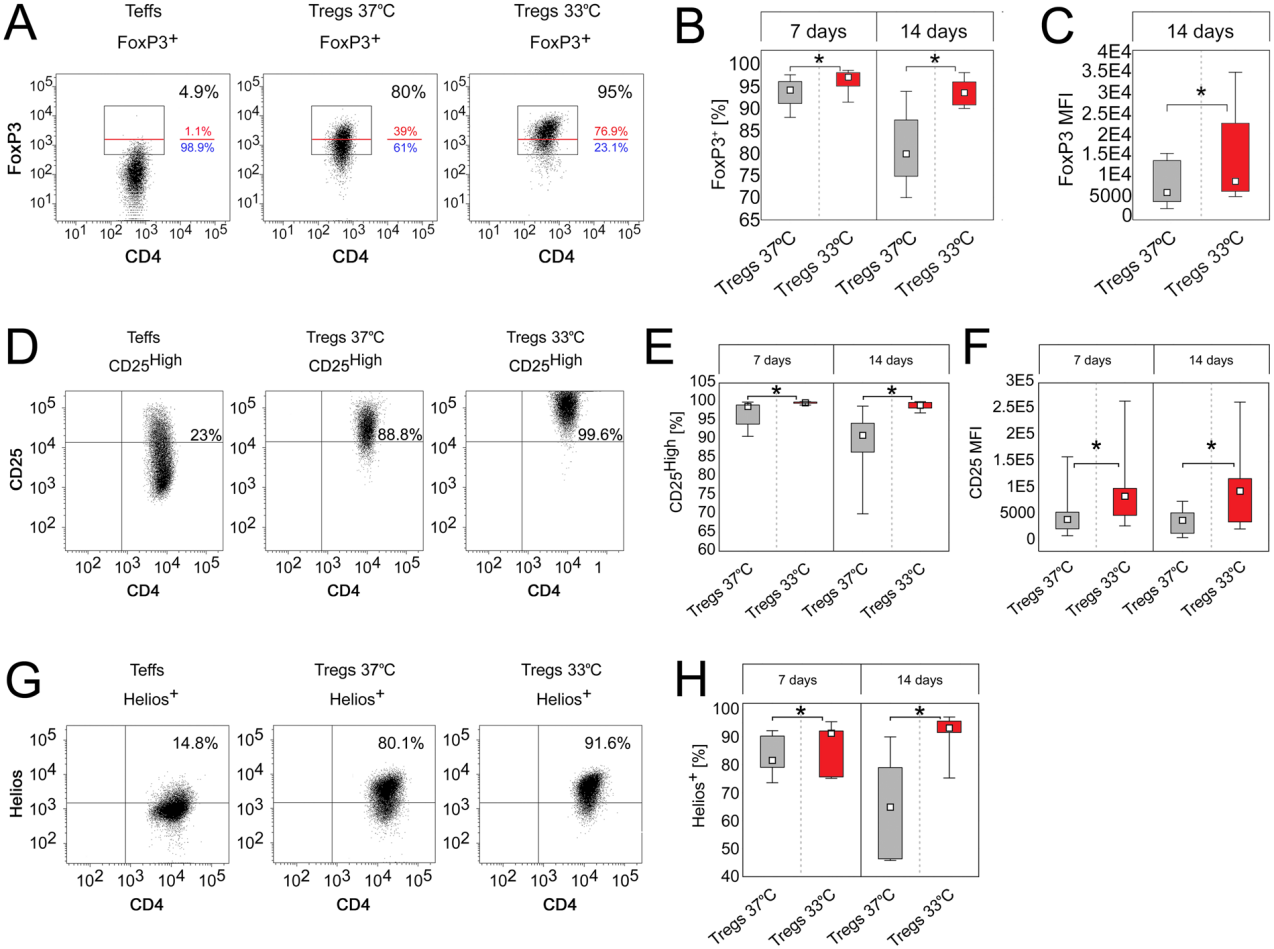
Temperature of 33 °C enhances expression of FoxP3, CD25 and Helios in Tregs. (**A**) Representative dot plots depicting FoxP3 expression in Tregs cultured at 37 and 33 °C for 14 days. CD4^+^ Teffs derived from the same donor and expanded at 37 °C are shown as a control. FoxP3^+^ cells are gated and their frequency within CD4^+^ population is shown in the right upper corner of each dot plot. Red line divides FoxP3^+^ population into FoxP3^High^ and FoxP3^Low^ cells which proportions are depicted with red and blue font, respectively. (**B**) Frequency of FoxP3^+^ cells within CD4^+^ population on day 7 and 14 of Treg expansion at 37 and 33 °C (gray and red symbols, respectively; n = 11). (**C**) Intensity of FoxP3 expression (MFI) in CD4^+^FoxP3^+^ cells after 14-day expansion at 37 and 33 °C (gray and red symbols, respectively; n = 11). (**D**) Representative dot plots depicting CD25 expression in Tregs cultured at 37 and 33 °C for 14 days. CD4^+^ Teffs derived from the same donor and expanded at 37 °C are shown as a control. CD25^High^ cells are gated and their frequency is shown in the right upper corner of each dot plot. (**E**) Frequency of CD25^High^ cells within CD4^+^FoxP3^+^ population on day 7 and 14 of Treg expansion at 37 and 33 °C (gray and red symbols, respectively; n = 11). (**F**) Intensity of CD25 expression (MFI) by CD4^+^FoxP3^+^CD25^High^ cells after 7 and 14-day expansion at 37 and 33 °C (gray and red symbols, respectively; n = 11). (**G**) Representative dot plots depicting Helios expression in Tregs cultured at 37 and 33 °C for 14 days. CD4^+^ Teffs derived from the same donor and expanded at 37 °C are shown as a control. Helios^+^ cells are gated and their frequency is shown in the right upper corner of each dot plot. (**H**) Percentage of Helios^+^ cells within CD4^+^FoxP3^+^ population at day 7 and 14 of Treg expansion at 37 and 33 °C (gray and red symbols, respectively; n = 5). MFI is presented as value to power of 4 or 5 (e.g. 1E5 = 1 × 10^5^). Statistical differences (**B-C,E-F,H**) were calculated with Mann- Whitney U test and are presented as medians (min.-max.). *p < 0.05.

Tregs cultured at 33 °C were characterized by significantly higher frequency of FoxP3^+^ T cells on day 7 and 14 (MW, p = 0.01 and p = 8 × 10^−5^, respectively) of the expansion than those cultured at 37 °C (Fig. 2A,B). FoxP3 expression was also significantly more stable over time in cells expanded at 33 °C. There was a 13.2% (median) decrease in frequency of FoxP3^+^ cells between day 7 and 14 when Tregs were expanded at 37 °C, while only 2% (median) decrease was observed at 33 °C (MW, p = 1 × 10^−4^; Table 1). In addition, since day 7 an escalating trend towards higher intensity of FoxP3 production by FoxP3^+^ cells was observed at 33 °C and on day 14 FoxP3^+^ cells expanded at 33 °C produced on average ≈2 fold more FoxP3 than corresponding Tregs at 37 °C (MW, p = 0.02; Fig. 2C).

**Table 1:** Stability of Treg phenotype at 37 and 33 °C.

		Tregs expanded at 37 °C		Tregs expanded at 33 °C		Difference
		median	min → max	median	min → max	p
Difference between day 7 and 14 of the culture	% of FoxP3^+^ cells within CD4^+^ population	−13.2	−26 → −3.3	−2	−8 → + 0.4	1 × 10^−4^*
	% of FoxP3^High^ cells within CD4^+^FoxP3^+^ population	−22.3	−57.2 → −1.7	−13.2	−28.9 → + 1.4	0.04*
	% of Helios^+^ cells within CD4^+^FoxP3^+^ population	−8.7	−45.8 → −0.1	+ 1.1	+ 0.1 → + 15.9	7 × 10^−3^*
	% of CD25^High^ cells within CD4^+^FoxP3^+^ population	−7.9	−20.7 → −0.2	− 0.55	−2.6 → + 0.2	2 × 10^−3^*
	% of CD25^High^ cells within CD4^+^FoxP3^High^ population	−3.2	−17.9 → + 1	−0.3	−0.9 → + 0.2	0.02*
	% of cells with demethylated TSDR	−24.5	−45.9 → −6.6	−5.5	−13.74 → + 0.37	0.05

The table presents differences in Treg phenotype between day 7 and 14 of the culture for cells expanded at 37 and 33 °C. To calculate the differences, results for day 7 were subtracted from results for day 14 and thus “−” and “+” mean decrease and increase of analyzed value over time, respectively. The data are results of 11 independent experiments, with the exception for Helios expression and TSDR demethylation where n = 5. The differences were calculated with Mann-Whitney U test, *p < 0.05.

When expression of CD25 was analyzed, we found that nearly 100% of CD4^+^FoxP3^+^ Tregs expanded at 33 °C were simultaneously CD25^High^ cells for entire duration of the culture, while frequency of CD25^High^ cells within CD4^+^FoxP3^+^ population was significantly lower at 37 °C (MW, p = 2 × 10^−4^ on day 7 and 14; Fig. 1D,E). In addition, CD4^+^FoxP3^+^CD25^High^ Tregs cultured at 33 °C produced significantly more CD25 than corresponding cells at 33 °C (MW, p = 0.01 on day 7and 14; Fig. 1F). Further analysis revealed that expression of CD25 was more stable in Tregs cultured at 33 °C than those at 37 °C (MW, p = 2 × 10^−3^; Table 1).

Analogously, frequency of Helios^+^ cells within CD4^+^FoxP3^+^ population was significantly higher at 33 °C (median = 93.2%) on day 14 than at 37 °C (median = 64.9%, MW, p = 0.03; Fig. 1G,H). When % of Helios^+^ cells on day 7 and 14 was compared, we found stable Helios expression only at 33 °C (Table 1, Fig. 1H). No differences in intensity of Helios expression (MFI) in CD4^+^FoxP3^+^Helios^+^ cells were observed when Tregs expanded at 33 and 37 °C were compared.

**Figure 3:**
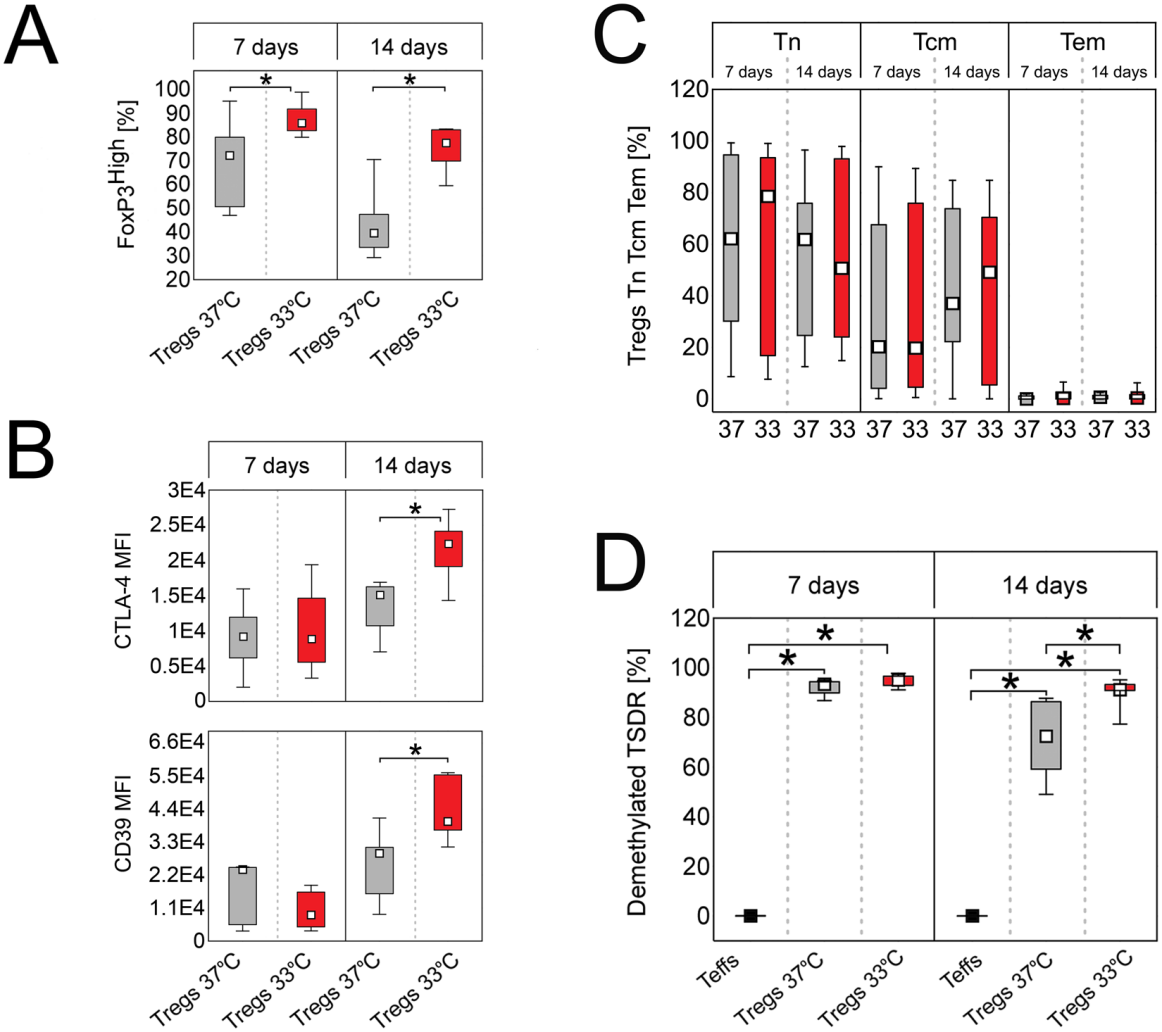
Tregs expanded at 33 °C are more stable and express higher amounts of Treg associated markers, than the cells cultured at 37 °C despite the same proportions of naive and memory subsets. (**A**) Frequency of FoxP3^High^ cells within CD4^+^FoxP3^+^ population on day 7 and 14 of Treg expansion at 37 and 33 °C (gray and red symbols, respectively; n = 11). (**B**) Intensity of CTLA-4 and CD39 expression (MFI) by CD4^+^FoxP3^+^CTLA-4^+^ and CD4^+^FoxP3^+^CD39^+^ cells, respectively after 7 and 14-day expansion at 37 and 33 °C (gray and red symbols, respectively; n = 7). MFI is presented as value to power of 4 (e.g. 1E4 = 1 × 10^4^). (**C**) Proportions of naive (Tn), central memory (Tcm) and effector memory (Tem) cells within CD4^+^FoxP3^+^ population at 37 and 33 °C on day 7 and 14 of the culture (n = 11). (**D**) Frequency of cells with demethylated *FOXP3* TSDR on day 7 and 14 of Treg expansion at 37 and 33 °C (gray and red symbols, respectively; n = 5). CD4^+^ Teffs derived from the same donors (black symbols, n = 5) and expanded at 37 °C are shown as a control. The statistical differences (**A–D**) were calculated with Mann-Whitney U test and are presented as medians (min.-max.). *p < 0.05.

In addition, Tregs cultured at 33 °C comprised significantly more FoxP3^High^ cells at day 7 (MW, p = 1 × 10^−3^, median = 86%) and 14 (MW, p = 2 × 10^−5^, median = 77.6%) than Tregs expanded at 37 °C (median, 72.3% and 39.6%, respectively; Figs 2A and 3A). Noteworthy, significantly more CD4^+^FoxP3^High^ cells had also phenotype of CD25^High^ cells when expanded at 33 °C, as compared with the corresponding population at 37 °C at both control points (MW, day 7 p = 1 × 10^−3^, day 14 p = 2 × 10^−3^; Figure S1A). CD4^+^FoxP3^High^D25^High^ cells expanded at 33 °C expressed also more CD25 than those cultured at 37 °C (MW, day 7 p = 0.01, day 14 p = 0.04; Figure S1B). Analogously to FoxP3^+^ cells, FoxP3^High^ population was more stable at 33, than at 37 °C. Tregs expanded at 33 °C were characterized by significantly lower decrease in number of CD4^+^FoxP3^High^ and CD4^+^FoxP3^High^CD25^High^ cells during culture *in vitro*, than those expanded at 37 °C (MW, p = 0.04 and p = 0.02, respectively; Table 1).

When other Treg associated markers were analysed, we found that nearly 100% (median ≥99%) of Tregs were positive for CD39 and CTLA-4 during entire culture *in vitro* regardless culture temperature. In addition, intensity of CTLA-4 and CD39 expression was found to increase with time at both studied conditions (MW, day 7 vs day 14 at 37 and 33 °C p ≤ 0.05, Fig. 3B). However, Tregs expanded at 33 °C were characterized by significantly higher expression of CTLA-4 (MW, p = 0.01) and CD39 (MW, p = 8 × 10^−3^) on day 14, than cells cultured at 37 °C (Fig. 3B). In addition higher expression of CTLA-4 and CD39 corresponded with greater production of FoxP3 on day 14 (Spearman’s rank correlation, SRC; R = 0.75, p = 4 × 10^−3^ and R = 0.6,p = 0.03, respectively). Frequencies of Nrp-1^+^ Tregs at both studied culture conditions were comparable and low (on day 7 and 14 median <5%). These results are in accordance with study of Battaglia *et al*. who showed that CD4^+^Nrp1^+^ T cells in human peripheral blood are exceedingly rare and most of them do not express Treg markers CD25 and Foxp3^21^. In addition, no statistically significant differences were found for intensity of Nrp-1 expression by Nrp-1^+^ Tregs expanded at 33 and 37 °C (data not shown).

Unexpectedly, Tregs cultured at 33 and 37 °C did not differ in proportions of naive (Tn, CD4^+^FoxP3^+^CD45RA^+^CD62L^+^), central memory (Tcm, CD4^+^FoxP3^+^CD45RA^-^CD62L^+^) and effector memory (Tem, CD4^+^FoxP3^+^CD45RA^-^CD62L^-^) cells on day 7 and 14 of the expansion (Fig. 3C). In addition, after 14-day culture median/mean frequency of all CD45RA^+^ cells within CD4^+^FoxP3^+^ population at 37 °C and 33 °C was equal to 62.8/56.5% and 50.7/54.8%, respectively.

As hypothermia is associated with cold stress, we aimed to analyse expression of heat shock proteins (HSPs) −60, −70 and −90 that are molecular chaperones known to protect cells from cellular and environmental stress factors^22^. However, no differences in intracellular expression of HSP-60, −70 and −90 (Figure S2), as well as their gene transcription (Table S1) were found between Tregs expanded at 33 and 37 °C. No correlations between expression of HSPs and FoxP3 or other Treg related markers were found. However, in both studied culture conditions we observed a significant increase in magnitude of HSP-60, −70 and −90 expression during culture *in vitro* as compared with day 0 (data for Tregs before their isolation form peripheral blood mononuclear cells, PBMC, Figure S2).

In contrast to cells expanded at 33 °C, Tregs cultured at 29 °C were found to lose their characteristic phenotype dramatically fast (Figure S3A). Between day 7 and 14 on average a 64% decrease in frequency of FoxP3^+^ cells was observed at 29 °C (median % of FoxP3^+^ cells on day 7 and 14 was 79.3% and 14.8%, respectively). On day 14 32.5% and 76.7% (median) of FoxP3^+^ cells remained CD25^High^ and Helios^+^, respectively at this condition.

Unlike 29 °C, temperature of 39 °C did not affect Treg phenotype significantly. After 14-day culture at 39 °C frequency of FoxP3^+^ cells was 79.8%, while CD25^High^ cells constituted 83.4% and 74.3% of CD4^+^FoxP3^+^ and CD4^+^ populations, respectively (Figure S3B).

In 2/13 cultures where Treg proliferation was significantly decreased at 37 °C the cells were losing their characteristic phenotype dramatically fast between day 7 and 14. In these cultures 3.3 and 6.2% of cells remained FoxP3^+^ on day 14 at 37 °C, while in parallel cultures at 33 °C frequencies of FoxP3^+^ cells were 90.8 and 92.8%, respectively. However, these results were excluded from general data analysis as significant deviation.

### Expansion at 33 °C prevents TSDR methylation during culture *in vitro*

Tregs expanded at 33 °C were characterized by significantly higher frequency of cells with demethylated TSDR after culture *in vitro*, than those at 37 °C. The differences have been escalating with time and reached statistical significance on day 14 (MW, p = 0.03; Fig. 3D). Notably, TSDR demethylation was kept stable over time only in Tregs cultured at 33 °C (Fig. 3D and ﻿Table 1).

**Figure 4:**
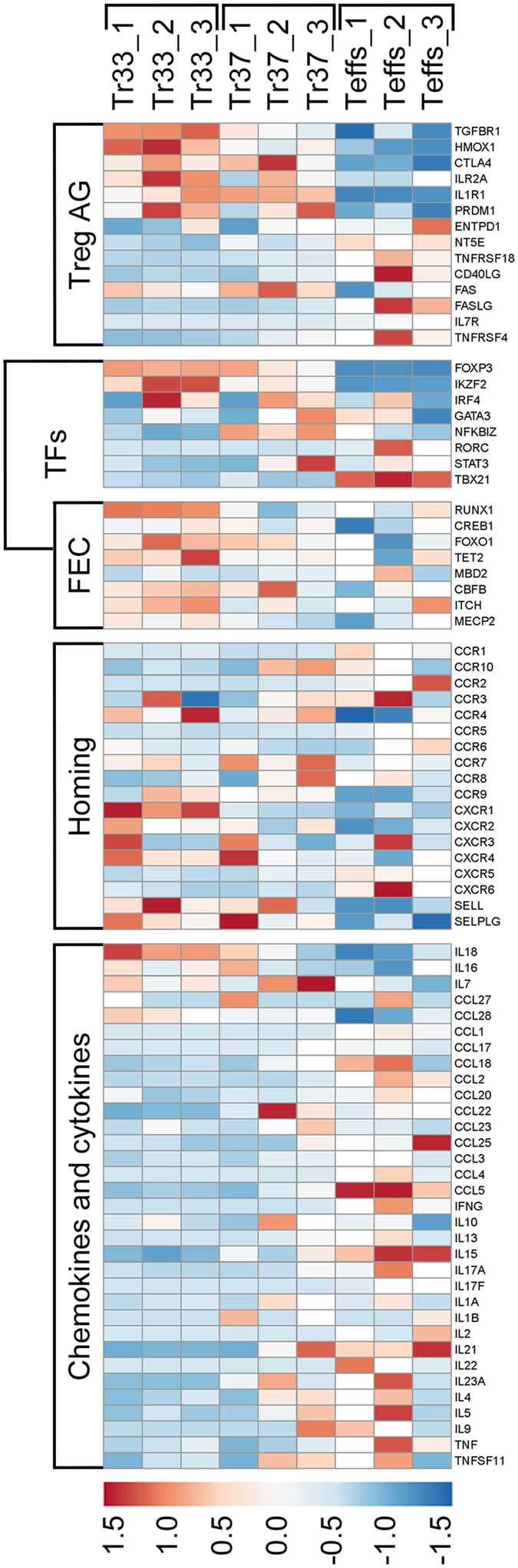
Tregs expanded at 33 °C present remarkably anti-inflammatory Treg-specific phenotype. Expression of selected: Treg associated genes (Treg AG), transcription factors (TFs), FoxP3 expression controlling genes (FEC), genes encoding receptors associated with cell migration (Homing) and genes for multiple chemokines and cytokines (Chemokines and cytokines) in 14-day cultures of Tregs expanded at 33 °C (Tr33, n = 3) and 37 °C (Tr37, n = 3) are shown. Results for CD4^+^ Teffs (Teffs, n = 3) derived from the same donors and expanded for 14 days at 37 °C are shown as a control. Relative FPKM values were used for generation of the heatmap.

When we analyzed expression of gens known to be involved in regulation of TSDR demethylation^20,23^ we found a trend towards enhanced transcription of *RUNX1* (log2 fold change = 0.54, p = 7 × 10^−3^, q = 0.24) and *TET2* (log2 fold change = 0.34, p = 0.1, q = 0.74) in Tregs expanded at 33 °C, while *MBD2* and *CBFB* were expressed at the same levels at both studied temperatures (log2 fold change = −0.02, p = 0.9, q = 0.99 and log2 fold change = 0.04, p = 0.81, q = 0.99, respectively; Fig. 4; Table S1).

Higher frequency of cells with demethylated TSDR within Tregs expanded at 33 °C did not result from lower contamination with non-Th cells. Post-expansion analysis with flow cytometry showed that at both culture temperatures CD56^+^, CD16^+^, CD19^+^ and CD14^+^ cells constituted on average ≤ 0.5%, while CD8^+^ lymphocytes ≤2.5% of total population and up to 100% of CD8^+^ cells were also positive for CD4. Presence of double-positive T cells in Treg cultures was also reported by other group working on clinical application of Tregs^9^. In addition, RNA-seq revealed comparable amounts of transcripts for *CD8A*, *CD8B*, *CD14*, *NCAM1*/*CD56*, *MS4A1/CD20*, and *FCGR3A/CD16a* (Table S1) in Tregs expanded at 37 and 33 °C.

### Tregs expanded at 33 °C produce less IFN-γ but are not significantly more potent inhibitors of IFN-γ production by Teffs than Tregs at 37 °C

After 14-day culture 2.5% and 4.5% (median) of Tregs were IFN-γ^+^, when cultured at 33 and 37 °C, respectively (MW, p = 7 × 10^−3^; Figure S4A). The results were concordant with RNA-seq data that revealed significantly lower abundance of *IFNG* transcript in Tregs at 33 °C, than at 37 °C (log2 fold change = −3.5, p = 5 × 10^−5^, q = 6 × 10^−3^; Table S1).

No statistically significant differences between Tregs expanded at 33 and 37 °C where found when we compared their capability for suppression of IFN-γ production by Teffs. Nevertheless, a slight trend towards stronger suppression by Tregs expanded under hypothermic conditions was observed (Figure S4B).

### Tregs cultured at 33 °C inhibit Teff proliferation more efficiently than Tregs expanded at 37 °C

**Figure 5:**
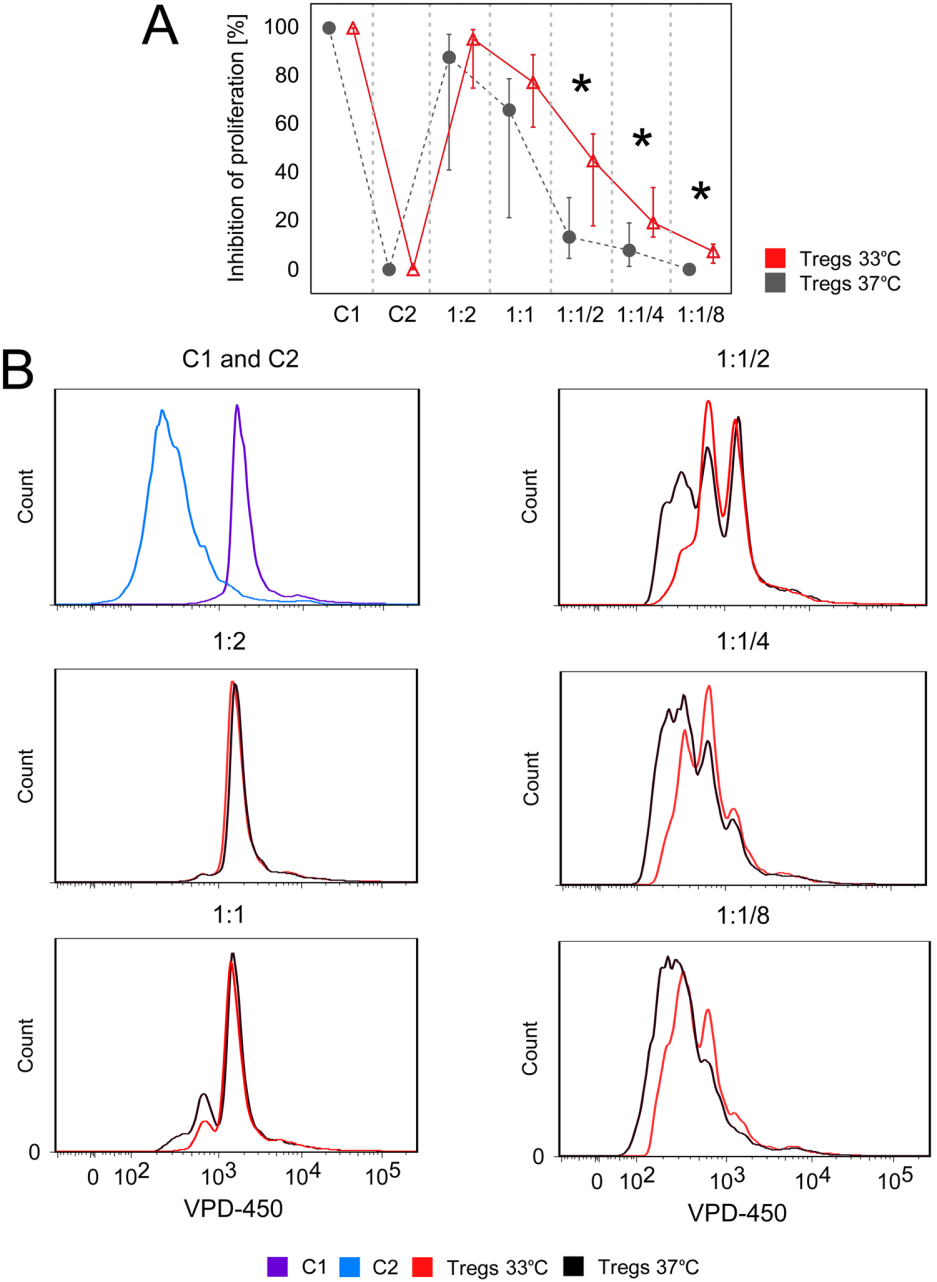
Tregs expanded at 33 °C are more potent inhibitors of Teff proliferation than Tregs cultured at 37 °C. (**A**) % of inhibition of Teff proliferation by Tregs expanded at 37 and 33 °C (gray and red symbols, respectively; n = 5). Results for various Teff:Treg ratios are shown. C1 = control unstimulated Teffs cultured without Tregs, value corresponds to complete inhibition of Teff proliferation (100%). C2 = control stimulated Teffs cultured without Tregs, value corresponds to complete lack (0%) of inhibition of Teff proliferation. (**B**) Histograms from 1 representative experiment are shown. Violet and blue histograms depict fluorescence of C1 and C2 controls, respectively. Inhibitory effect of Tregs expanded at 37 and 33 °C is reflected with black and red histograms, respectively.

Tregs cultured at 33 °C were found to suppress proliferation of Teffs more efficiently than those expanded at 37 °C. The effect was more pronounced for lower Treg:Teff ratios: ½:1, ¼:1 and ^1^/8:1 (MW, p = 0.03, p = 0.03 and p = 0.02, respectively; Fig. 5).

### Tregs expanded at 33 °C present remarkably anti-inflammatory phenotype

According to RNA-seq data analysis Tregs expanded at 33 and 37 °C significantly differed in expression of 238 genes (Table S1). Only 43 out of 238 genes which expression was significantly changed at 33 °C (p and q values < 0.05) were found to be upregulated in cells expanded at 33 °C, including 17 transcripts (namely *CYP4F3, GRM4, GRM7, LRRIQ4, PCDHB8, PDLIM4, STEAP2, ADCYAP1R1, ACVRL1, ADORA1, ASTN1, PLCB4, KCNJ3, ZNF385B, NRG1, NEU4, KCNIP3*) that were detectable only in Tregs cultured at this temperature. Those with the most remarkable increase (log2 fold change > 0.7) included: *IKZF2* (Helios encoding gene), *HMOX1, IQGAP2, CDKN2A, CREG2, CXCR1, EXOC6B, PELI2, E2F2, GPR55, HIPK2, HPGD, ITM2C, PHTF2, PCDH8, RBM3, RDH10, KIAA1671, RFX7, SAMD3, SEMA3G, SLC7A11, TRAK2 and UBL3* (Table S1 and Fig. 4).

At the same time 195 genes were significantly (p and q values < 0.05) downregulated at hypothermic conditions and for 7 of them (*CCL17, IL9, BFSP1, HTR3B, SPIB, IRX5* and *WNT5A*) no mRNA was detected at 33 °C, while they were transcribed at 37 °C (Table S1). Besides, transcripts of other 945 genes (e.g. *IL1B, IL17REL, CCL14*) were not detectable in Tregs expanded at 33 °C but at 37 °C. Although those differences were not statistically significant, they clearly show that temperature of 33 °C suppresses transcription of ≈1000 genes in Tregs, while their expression at 37 °C is promiscuous (Table S1).

In general, downregulated genes at 33 °C were associated with cell activation (e.g. *HLA-DR, HLA-DQ, CD40LG/CD40L, NFKB2, TNFRSF4/OX40, CD69, ICAM1*)^24^, Th-1 and Th-17 responses (e.g. *IFNG, IL1A, TNFRSF25, TNFSF11/RANKL, IL17RB, THY1/CD90, KLRB1/CD161, NFKBIZ*)^25–28^, Th-2 cell activation (e.g. *IL13, IL9, GATA3-AS1, CCL17*)^29–31^ and basically with proinflammatory response (e.g. *CCL3/MIP-Iα, CCL4/MIP-Iβ, CCL5/RANTES*)^32^ underling anergic character of Tregs expanded at 33 °C (Fig. 4, Table S1).

To get insight into the biological context of differentially expressed genes in our dataset we made core analysis in Ingenuity Pathway Analysis (IPA) software. Disease and function mode resulted in the list of 155 phenomena that were inferred to be significantly more downregulated and/or inhibited (z-score ≤−2, p-score < 0.05) by Tregs cultured at 33 °C, than by those at 37 °C. Ten functions with the lowest activation z-score (the most potent inhibition) are listed in Table 2, and strongly correlate with anti-inflammatory profile of Tregs expanded at hypothermic conditions. Regulator effects algorithm implemented in IPA showed the association between our dataset molecules, the upstream regulators and downstream effects. The corresponding regulatory network with the highest consistency score is presented in Figure S5.

**Table 2:** Top ten functions and diseases predicted to be altered by Tregs expanded at 33 °C in comparison with cells cultured at 37 °C.

	Disease/function	p-score	Activation z-score ^a^
1	activation of cells	3.18 × 10^−13^	−4.643
2	development of mononuclear leukocytes	2.55 × 10^−13^	−4.077
3	generation of cells	1.54 × 10^−13^	−4.03
4	activation of leukocytes	9.87 × 10^−13^	−4.027
5	differentiation of cells	3.56 × 10^−16^	−3.942
6	migration of cells	3.18 × 10^−14^	−3.919
7	size of body	1.29 × 10^−6^	−3.881
8	recruitment of leukocytes	2.3 × 10^−11^	−3.87
9	development of lymphocytes	9.99 × 10^−13^	−3.819
10	cell movement	1.79 × 10^−15^	−3.792

The table shows the top 10 Ingenuity Pathway Analysis (IPA) functions and diseases affected by Tregs expanded at 33 °C based on differentially expressed genes in these cells as compared with Tregs cultured at 37 °C.

^a^Algorithm in IPA designed to reduce the chance that random data will produce significant prediction. It identifies functions with the strongest prediction for increase (positive z-score) or decrease (negative z-score). Values of p-score <0.05 and z- score ≤−2 or ≥2 are considered significant.

## Discussion

Temperature of 37 °C has been established the optimal temperature for human and animal cell cultures for years. As it is considered a physiological temperature of human body, none controverted that Tregs should be expanded at 37 °C. Nevertheless, in the current paper we showed for the first time that optimal temperature for Treg culture is lower than currently established standard. We demonstrated that mild hypothermia of 33 °C preserves Treg phenotype and function during culture *in vitro* and makes Treg expansion more predictable, consistent and effective, than it was reported before.

Multiple previous studies, including ours suggested that natural Tregs down-regulate FoxP3 expression after repetitive stimulation *in vitro* and convert into non-regulatory cells in time-dependent fashion^4,12–14,16^. Then, Hoffmann *et al*. also reported that high FoxP3 expression and immunosuppressive potential of Tregs can be kept for 2-3-weeks *in vitro* when CD4^+^CD25^High^CD45RA^+^ Treg subset is selected for expansion. Despite CD45RA^+^ Tregs were found to rapidly convert into CD45RA^−^ cells during the culture, they presented more stable Treg phenotype and function after expansion, than Tregs derived from CD45RA^-^ or mixed CD45RA^+^ and CD45RA^-^ populations. Therefore, it was suggested that only CD45RA^+^ expressing Tregs can give rise to stable and homogenous T cell lines after expansion *in vitro* ^12,33^. Nevertheless, in our current study the sorted Treg populations each time comprised both CD45RA^+^ and CD45RA^-^ cells. Despite this initial cell heterogeneity, only Tregs expanded at 33 °C remained stable, regardless presence or absence of CD45RA. Moreover, frequencies of CD45RA^+^ cells, as well as proportions of naive and memory subsets identified according to expression of CD45RA and CD62L^7,34^ were comparable in both culture conditions after expansion. Thus, CD45RA does not seem to be vital for Treg function and stability. These observations are in accordance with our recently published study that showed that CD45RA^−^ Tregs can express high levels of FoxP3, have demethylated TSDR and are crucial for graft tolerance^35^. All together these data call into question the previously accepted dogma and indicate that Treg phenotype and function *in vitro* and *in vivo* does not depend on CD45RA.

Soon after discovery of Tregs it was suggested that they lose immunosuppressive activity during proliferation^36^. However, in our study Tregs cultured at 33 °C exhibited more potent immunosuppressive potential and simultaneously proliferated 4.5-fold more efficiently, than Tregs at 37 °C. This high proliferative potential can at least partially result from increased expression of α-chain of high-affinity receptor of IL-2 (CD25/ILR2A) on Treg surface at 33 °C. Two functional IL-2 receptors (IL2R) were described, the dimeric low-affinity receptor composed of CD122 (IL2RB) and CD132 (IL2RG) and trimeric high-affinity receptor consisting of CD122, CD132 and CD25^37^. Upregulation of CD25 could increase proportion of high-affinity to low-affinity IL2Rs on Tregs at 33 °C. IL-2 is a known mitogen of T cells and factor required for Treg survival and proliferation^37^. Therefore, enhanced production of CD25 and thus upregulation of high affinity IL2R on Tregs at 33 °C could result in higher IL-2 responsiveness and proliferation. In addition, it is known that due to presence of high-affinity IL2R Tregs consume large amounts of IL-2 and thus deprive other T cells of the cytokine^37^. This mechanism, at least partially, could be responsible for stronger suppression of Teff proliferation by Tregs expanded at 33 °C in our study.

During our 12-year experience with *ex vivo* expansion of human Tregs we found that Tregs of some patients do not proliferate *in vitro*. Generally, these cells remain FoxP3^+^ but in some cases, like in the present study, they began to lose FoxP3 expression dramatically fast after the first week of the culture. This Treg proliferation arrest is not strictly related to donor age, however it is more frequent when cells derive from older individuals. We observed it in 3/100 expansions of Tregs from diabetic children and in the present study in 2/13 cultures at 37 °C (cells from healthy adult donors). Our experience is not unique. Theil A. and her group also observed that Tregs from some adult donors (2/12 samples) present very low proliferation rate, thus no clinically optimal Treg dose can be obtained (Theil A., personal communication at meeting of Action to Focus and Accelerate Cell-based Tolerance-inducing Therapies, Galway 2016). Brunstein CG *et al*. have also reported recently that 2/13 clinical Treg expansions failed to achieve the target cell dose due to low Treg proliferation^10^. This phenomenon is a serious problem for clinical Treg therapy. However, decreasing culture temperature to 33 °C we could overcome this obstacle. In our study Tregs that did not expand at 37 °C and lost FoxP3 expression after 14-day culture, remained FoxP3 positive (>90%) and proliferated extensively, when they were kept at 33 °C. Thus, a single and simple change in culture conditions can be a solution for this problem.

Our RNA-seq data and TSDR methylation analyses suggest that mild hypothermia not only stimulates Treg proliferation, but also induces different transcriptional program and influences epigenetic modifications that together provide Treg stability. Recent studies suggested that TSDR demethylation results, at least partially, from action of runt-related transcription factor 1 (Runx1) and core-binding factor subunit β (Cbf-β) that shield TSDR from methylating enzymes. These molecules were also shown to be required for Foxp3 mRNA and protein expression in Tregs^20,23^. In addition, it was demonstrated that TSDR demethylation in Tregs is regulated by DNA demethylase Tet2 (tet methylcytosine dioxygenase 2) that acts in cooperation with Mbd2 (methyl-CpG-binding domain protein 2)^20,38,39^. When we analyzed transcription of genes encoding these proteins in our samples, we found a pronounced trend towards enhanced expression of *RUNX1* and *TET2* in Tregs expanded at 33 °C as compared with those cultured at 37 °C. No differences were found for *CBFB* and *MBD2* mRNA levels. These data suggest that Runx1 and Tet2 dependent pathways can be involved in stable TSDR demethylation in Tregs at hypothermic conditions. In addition, previous studies showed that FoxP3-Runx1 complex represses *IL2* and *IFNG* genes, and at the same time activates *IL2RA/CD25* expression^40^. These data are consistent with our observations. Tregs expanded at 33 °C were characterized by higher transcription of *Runx1* and *FoxP3*, as well as expression of FoxP3 protein and thus contained significantly less mRNA of *IFNG* and presented higher surface expression of CD25, than those at 37 °C. In neither of Treg samples transcripts of *IL2* were detected.

Another important issue associated with effective Treg function is their stability under inflammatory conditions. It was reported that Helios (IKAROS family zinc finger 2; IKZF2) renders Tregs anergic and suppressive even during intense inflammatory response. At the same time, Helios-deficient Tregs were found to be characterized by reduced expression of FoxP3, diminished lineage stability and secretion of proinflammatory cytokines. Recently it was even suggested that deletion of Helios promotes Treg conversion into Teffs^41^. In our study, only Tregs expanded at 33 °C were found to keep Helios expression at the same level for the entire culture duration, while its time dependent decrease was observed in Tregs at 37 °C. Stability of Tregs expanded at 33 °C was also confirmed by TSDR demethylation analysis. Only Tregs cultured at hypothermic conditions kept their TSDR demethylated throughout the culture *in vitro* and were significantly more potent inhibitors of Teff proliferation.

All these observations shed new light on impact of temperature on immune system, the issue that was thought to be completely explored. Fever and associated changes in immune response have been extensively studied many years ago^42^ and it seemed that the knowledge in this field was complete. However, our study provides fresh insight into biological sense of temperature changes. Fever is one of the most common symptoms of infection and signal for immune system activation. Bearing in mind that Tregs suppress immune responses and participate in their termination^43^, we should expect that fever deteriorates Treg function. However, once pathogen is eradicated, normothermia regained, Tregs should activate to prevent prolonged and potentially harmful immune response. Bearing in mind these interrelationships, increased Treg activity, stability and proliferation at lower temperature of 33 °C is not surprising. For effective immune defense Tregs need to be inhibited as long as pathogen is present and fever is established. Thus, high temperatures should be expected to suppress and low temperatures to stimulate Treg proliferation and/or function. Our observations confirm this hypothesis. Only Tregs expanded at mild hypothermia were characterized by high and stable expression of FoxP3, Helios and CD25, as well as robust proliferation. In addition, 13/13 of Treg samples expanded at 33 °C vs 1/13 at 37 °C contained >59% of FoxP3^High^ cells within FoxP3^+^ pool after 14-day expansion, while the amount of FoxP3 protein in Tregs correlates with their suppressor function^44,45^. Besides, Tregs cultured at 33 °C presented profound anti-inflammatory profile and no transcripts of *IL1B, IL17REL, CCL17* and *IL9* were detected in these cells, in contrary to Tregs at 37 °C. Transcription of several other genes involved in immune cell activation such as *IL1A/IL1α, CD40LG/CD40L, TNFRSF4/OX40*, *NFKB2*, *IFNG, TNFRSF25*, *TNFSF10*/*TRAIL, TNFSF11/RANKL, CCL3, CCL4, CCL5, IL17RB* and *IL13* was significantly downregulated at 33 °C as compared with the cells cultured at 37 °C. Concomitantly, mild hypothermia enhanced Treg expression of *HMOX1*, a gene encoding heme oxygenase 1 (HMOX1). It was reported previously that HMOX1 expression is induced by FoxP3 and is critical in FoxP3-mediated immune suppression. Choi *et al*. demonstrated that Tregs became unable to suppress Teff proliferation after blockage of HMOX1^46^. Subsequently, Brusko *et al*. proposed that Treg suppressive activity (inhibition of proinflammatory cytokine production by Teffs and Teff proliferation) depends on production of carbon monoxide (CO) via HMOX1 in activated Tregs^47^. Moreover, studies *in vivo* showed that induction of HMOX1 prolongs graft survival, while inhibition worsens the outcome of allo- and xenotransplantation. This effect was dependent on direct and indirect actions of HMOX1 that included nonspecific cytoprotection from oxidative damage, vasodilation, antiplatelet aggregation effect and inhibition of host inflammatory responses mediated by neutrophils, macrophages and lymphocytes^47,48^. Moreover, HMOX1 expression in donor and recipient tissues was shown to promote activation induced cell death of allorective lymphocytes after allograft transplantation^49^, while T cells transfected with HMOX1 were found to became resistant to Fas-mediated apoptosis^50^, suggesting that HMOX1 is important for both Treg function and survival. HMOX1 was also found to ameliorate various T cell mediated diseases due to its wide range of anti-inflammatory and immune regulatory mechanisms of action^51–54^. Inter alia, enhanced expression of HMOX1 in animal models of inflammatory bowel disease (IBD) and asthma was observed to attenuate symptoms of the diseases by inhibition of Th17 responses^51^. Moreover, Freitas *et al*. revealed that secreted metabolites of HMOX1 decreased leukocyte rolling, adhesion and neutrophil migration to the site of inflammation^54^. Thus, hypothermia induced upregulation of HMOX1 in Tregs can have a broad beneficial impact on immune tolerance induction after administration of these cells to patients.

In addition, Tregs expanded at 33 °C were characterized by higher expression of CTLA-4 and CD39 at the end of the culture. These two molecules contribute to Treg immunoregulatory function and play an important role in suppression of T cell activation and proliferation^1,55^. CTLA-4 is known to induce expression of indoleamine 2,3-dioxygenase (IDO) in dendritic cells (DCs) that suppresses T cell proliferation via depletion of tryptophan and accumulation of kynurenine^56^. While CD39 is an ectoenzyme involved in generation of pericellular adenosine that suppresses T cell function. Adenosine was also found to enhance generation of induced Tregs by inhibition of IL- 6 expression and promotion of TGFβ secretion^1,55^. Moreover, both CTLA-4 and CD39 favour generation of tolerogenic DCs^1,55,57^ that is of great importance for attenuation of deleterious immune response *in vivo*. All together these data show that hypothermia can have a broad impact on Treg activity and intercellular interactions. This way our study contributes to better understanding of beneficial effects of cryotherapy and regulation of the immune activation in general.

Prolonged hypothermia is associated with cold stress, therefore in the current study we measured intracellular expression and gene transcription of HSPs, phylogenetically conserved proteins known to protect the cells from cellular and environmental stress^22^. We hypothesized that hypothermia enhances expression of these molecules and that HSPs are responsible for preservation of Treg stability *in vitro*. Our theory was supported by previous studies of Vercoulen *et al*. and Zanin-Zhorov *et al*. who found that HSPs, notably HSP-60, can contribute to an increase in Treg number and function^23,58,59^. However, unexpectedly we found no alterations in intracellular expression of HSP-60, −70 and −90, as well as their gene transcription in Tregs expanded at 33 °C as compared with the cells at 37 °C. The only change in HSP expression in Tregs was observed when the cells at day 0 were compared with those expanded *in vitro*. However, the difference was the same for Tregs cultured at 33 and 37 °C. These data suggest that Treg transfer from peripheral blood to the artificial environment of the culture *in vitro* was a substantial stress that enhanced HSP expression, but mild hypothermia did not trigger this change. Thus, HSPs are not the crucial regulators of Treg response to temperature decrease.

In our study Tregs remained stable during culture *in vitro* at 33 °C, but the beneficial effect of hypothermia was kept also after transfer to 37 °C. All functional tests in our study were performed at 37 °C, as this is a physiological temperature of human body and *ex vivo* expanded Tregs are expected to function at this temperature *in vivo*. In these tests Tregs previously expanded at 33 °C revealed higher suppressive potential at 37 °C, than those cultured at this temperature since the beginning. Undoubtedly, full verification if Tregs expanded at 33 °C have better therapeutic effect *in vivo* requires further studies, but the presented data shed new light on regulation of Treg function. We may expect that activation status of Tregs expanded *ex vivo* will be altered after transfer to human body. However, Tregs expanded at 33 °C will start their function *in vivo* with highly demethylated TSDRs, increased FoxP3 expression, greater suppressive and proliferative potential, thus it is likely that they will exert better therapeutic effect. Application of hypothermia for Treg culture can be compared to trivial storage of food in fridge. Refrigeration, does not modify the structure of food, but enables longer storage of fresh products that do not compromise human health. In the same way delivery of fresh, stable and functional Tregs expanded at hypothermic conditions can be crucial for safety and effectiveness of Treg based therapies. Therefore, we have already started Treg production for clinical applications at 33 °C in the current clinical trials (TregVAC2.0EudraCT:2014-004319-35 and TregSM EudraCT: 2014-004320-22). Our preliminary results confirm safety and efficacy of this method of Tregs expansion.

In summary in the present study we showed for the first time that optimal temperature for Treg culture *in vitro* is lower than standard temperature of 37 °C. We demonstrated that mild hypothermia of 33 °C preserves Treg phenotype and function during culture *in vitro* and makes Treg expansion more predictable, consistent and effective, than it was reported before. Tregs expanded at 33 °C were found to be more potent inhibitors of Teff proliferation. Despite higher expansion rate they were characterized by more quiescent phenotype and lower production of proinflammatory cytokines, as compared with Tregs expanded at 37 °C. Higher Treg stability, effectiveness and proliferation at 33 °C makes this temperature optimal for *ex vivo* expansion of Tregs when their clinical application is planned and can be considered by teams who work on Treg based therapies.

## Methods

### Blood donors

Buffy coats were obtained from the Regional Centre for Blood Donation and Treatment in Gdańsk. All blood donors (n = 13) gave informed consent and all experimental protocols were approved by Independent Bioethics Commission for Research of the Medical University of Gdańsk (agreement no. NKEBN/353/2011). The study was conducted in accordance with guidelines of the Commission and Declaration of Helsinki.

### Treg and CD4^+^Teff isolation

Tregs and CD4^+^ Teffs were freshly isolated from buffy coats obtained from 13 volunteer blood donors according to our previously described protocol. Briefly, on the day of blood donation peripheral blood mononuclear cells (PBMC) were isolated from buffy coats by Ficoll/Uropoline gradient centrifugation and subjected to negative immunomagnetic selection (StemCell Technologies, Canada). Subsequently, CD4^+^ T cells were stained with monoclonal antibodies (mAb) specific for the following antigens: CD3, CD4, CD25, CD127, CD8, CD19, CD16 and CD14. The last 4 mAbs were conjugated with the same fluorochrome in aim to cut-off in one step cytotoxic T cells (Tc), B cells, natural killer (NK) cells and monocytes, respectively, that could potentially contaminate isolated CD4^+^ population. These cells were defined all together in sorting algorithm as *lineage*. Then, cells were sorted with FACS sorter Influx (BD Biosciences, USA) into the following phenotype of Tregs: CD3^+^CD4^+^CD25^High^CD127^−/Low^lin^−^doublet^-^ and Teffs: CD3^+^CD4^+^CD25^-^CD127^High^lin^−^doublet^-^ as it was reported before^4–7,14,60^.

### Cell expansion

After sorting Tregs were equally divided into 2 separate cultures marked as Tregs37 and Tregs33 and were expanded at 37 °C and 33 °C, respectively. In parallel sorted CD4^+^ Teffs were cultured at 37 °C, as they were used in further experiments and served as a control for Treg phenotype analysis. Besides difference in the culture temperature, other parameters of the expansion were the same for all cells. All Tregs and Teffs were expanded in penicillin (100 U/ml) and streptomycin (100 mg/ml) containing SCGM medium (CellGro, CellGenix, Germany) supplemented with 10% heat inactivated human AB serum, IL-2 (2 × 10^3^ U/mL, Aldesleukin, Chiron, USA) and magnetic beads coated with anti-CD3 and anti-CD28 antibodies (Invitrogen, USA) in a 1:0.6 cell:bead ratio. Beads were added to the cultures on day 0 and 7.

In the selected experiments size and granularity of Tregs37 and Tregs33 were analyzed daily with flow cytometry on basis of forward (FSC) and side scatter (SSC) parameters.

In aim to analyze dynamics of Treg proliferation in studied temperatures samples of Tregs37 and Tregs33 were stained with 5 μM of 5,6-carboxyfluorescein diacetate succinimidyl ester (CFSE; Vybrant® CFDA SE Cell Tracer Kit, Life Technologies, USA) for 15 min. at 37 °C on day 0 and CFSE fluorescence was measured daily. As complete loss of CFSE fluorescence was observed after 7 days due to intensive cell proliferation and consequent dye dilution, the cells were stained again after this period and restimulated. Strong polyclonal stimulation of Treg proliferation at 37 and 33 °C induced division cycle synchronization within each culture. Therefore, as it was reported before^61^, a progressive shift of a single peak of CFSE fluorescence was observed over time for each studied culture and their mean fluorescence intensities were analyzed to assess proliferation dynamics.

In 2 control experiments Tregs were cultured also at 29 °C and in a single experiment at 39 °C in aim to check an impact of significant change in standard culture temperature on Treg phenotype and proliferative capabilities.

Each time Tregs were cultured for 14 days and then harvested and counted after bead removal. The fold increase in cell number was calculated as final cell count on day 14 divided per initial cell number on day 0.

### Phenotype check

Each 7th and 14th day of the expansion samples of Tregs37, Tregs33 and Teffs were labeled with Abs against the following antigens (Ag): CD4, CD25, CD127, CD45RA (BD Biosciences, USA), CD62L (Life Technologies, USA), Nrp-1 (BioLegend, USA), HSP-60, HSP-70, HSP-90 (Abcam, UK), CD39, CTLA-4, Helios and FoxP3 using Foxp3 Staining Buffer Set (eBioscience, USA) and analyzed with flow cytometry (Canto II, BD Biosciences, USA). As it was reported before^7,34^ Tregs were assigned to naive (Tn), central memory (Tcm) and effector memory (Tem) populations according to the following phenotypes: CD4^+^FoxP3^+^CD45RA^+^CD62L^+^, CD4^+^FoxP3^+^CD45RA^-^CD62L^+^ and CD4^+^FoxP3^+^CD45RA^-^CD62L^−^, respectively.

### IFN-γ inhibition assay and measurement of IFN-γ production by Tregs

At 13th day of the expansion samples of Teffs were stained with CFSE (5 μM) for 15 min. at 37 °C and mixed with unstained Tregs37 or Tregs33 in the following proportions: 1:1, 1:^1^/_2_, 1:^1^/_4_ and 1:^1^/_8_. Cells were cocultured for the next 24 h at 37 °C (as it is considered a physiological temperature of human body) in SCGM medium supplemented with 10% heat inactivated human AB serum, IL-2 (100 U/ml), monensin, a protein transport inhibitor (GolgiStop, BD Biosciences; 2 μl/1000 μl of medium) and beads coated with anti-CD3 and anti-CD28 antibodies in a 1:1 Teff:bead ratio. In these experiments Teffs were stained with CFSE in aim to distinguish them from Tregs in cocultures during analysis of IFN-γ production. CFSE stained Teffs cultured alone in presence or absence of beads were used as controls. In addition, IFN-γ production by bead stimulated Tregs37 and Tregs33 cultured alone was also measured. After 24 h cells were harvested, labeled with anti-CD4 and anti-IFN-γ mAbs and analyzed with flow cytometer (Canto II, BD Biosciences, USA).

Data were analyzed as % of inhibition of IFN-γ production by Teffs. Results for all tested Teff:Treg proportions were analyzed in relation to two controls-unstimulated Teffs (C1) and bead-stimulated Teffs (C2), both cultured without Tregs. All data were converted in the same way, thus % of inhibition of IFN-γ production for unstimulated control Teffs was always equal to 100% and reflected lack of Teff activation and complete suppression of IFN-γ synthesis, while results for control stimulated Teffs were always equal to 0% and signified lack of inhibition of IFN-γ synthesis. Consequently, the more effective in suppression of IFN-γ production were Tregs, the higher % of inhibition of IFN-γ production was observed.

### Proliferation inhibition assay

At day 7 of the expansion a proliferation inhibition assay was performed. Teffs were stained with 2 μM of Violet Proliferation Dye 450 (VPD-450; BD Horizon, USA) for 15 min. at 37 °C and mixed with unstained Tregs37 and Tregs33 in the following Teff:Treg proportions: 1:2, 1:1, 1:^1^/_2_, 1:^1^/_4_, and 1:^1^/_8_. Cells were cocultured for the next 4 days at 37 °C in SCGM medium supplemented with 10% heat inactivated human AB serum and expanding beads in a 1:1 Teff:bead ratio. VPD-450 stained Teffs cultured alone in presence or absence of beads were used as controls. Just before the read-out, cells were labeled with 7-amino-actinomycin D (7-AAD, BD Pharmingen, USA) a compound that binds to DNA of nonviable cells in aim to exclude dead cells and then subjected to flow cytometry (Canto II, BD Biosciences, USA).

Data were analyzed as % of inhibition of Teff proliferation. Results for all tested Teff:Treg proportions were analyzed in relation to two controls-unstimulated Teffs (C1) and bead-stimulated Teffs (C2), both cultured without Tregs. All data were converted in the same way, thus % of inhibition of Teff proliferation for unstimulated control Teffs was always equal to 100% and reflected lack of Teff activation and complete suppression of their proliferation, while results for control stimulated Teffs were always equal to 0% and signified lack of inhibition of proliferation. Consequently, the more effective in suppression of Teff proliferation were Tregs, the higher % of inhibition of Teff proliferation was observed.

### DNA methylation of the Treg-Specific Demethylated Region (TSDR)

Genomic DNA from 7-day and 14-day cultures of Teffs, Tregs37 and Tregs33 was extracted with the QIAamp DNA blood mini kit (Qiagen, Hilden, Germany). A minimum of 60 ng bisulfite-treated (EpiTect; Qiagen) genomic DNA was used in a real-time PCR to quantify the Foxp3 Treg-specific demethylated region (TSDR). Real-time PCR was performed in a final reaction volume of 20 µl containing 10 µl FastStart universal probe master (Roche Diagnostics, Mannheim, Germany), 50 ng/µl lamda DNA (New England Biolabs, Frankfurt, Germany), 5 pmol/µl methylation or non-methylation-specific probe, 30 pmol/µl methylation or non-methylation-specific primers and 60 ng bisulfite-treated DNA or a respective amount of plasmid standard. The samples were analyzed in triplicates on a ABI 7500 cycler and reported as % of T cells with demethylated TSDR region. Treg samples with extremely low FoxP3 expression (2/13 cultures at 37 °C) were not subjected to these analyses as they were considered a significant deviation.

### Transcriptome analysis using massively parallel sequencing (RNA-seq)

RNA-seq was performed for 14-day cultures of Tregs37 and Tregs33. CD4^+^ Teffs from the same donors and expanded at 37 °C were analyzed as controls. Total RNA was isolated using RNeasy Mini Kit (Qiagen, Hilden, Germany), followed by quality and quantity assessment using 2100 Bioanalyzer (Agilent, Santa Clara, USA). RNA with a RNA Integrity Number (RIN) of 7.0 or above was used for sequencing library preparation. SureSelect Strand Specific mRNA library kit (Agilent, Santa Clara, USA) starting with two rounds of polyA selection was used followed by the standard procedure. The cDNA libraries were quantitated using qPCR in a Roche LightCycler 480 with the Kapa Library Quantification Kit for Illumina (Kapa Biosystemks, Woburn, USA) prior to cluster generation. mRNA-sequencing was performed on the Illumina HiSeq 2500 using the Rapid Run v2 sequencing chemistry and flow cells as described by the manufacturer (Illumina Inc., San Diego, USA). Cluster generation was performed according to the manufacturers recommendations for onboard clustering. Paired end 50 bp sequencing runs were completed and the data were converted to the FASTQ Sanger format using FASTQ Groomer. TopHat was used to align RNA-Seq reads to hg19 reference genome using the short-read aligner Bowtie^62^. SAM files were directly used as input in Cuffdiff 2.2.1^63^ implemented in Galaxy 2.2.1.3^64^ to look for significant changes in transcript expression in Tregs37 and Tregs33 with three replicates for each condition. We used quartile normalization method for the libraries, per-condition dispersion method for replicates and biased correction using canonical hg19 as reference. Additionally, differentially expressed genes were grouped in functional pathways with Ingenuity Pathway Analysis software (IPA Fall Release 2016, Redwood City, USA). Fisher’s exact test was implemented to measure the likelihood that the association between the differentially expressed genes and a given pathway is not due to random chance.

### Statistical analysis

Flow cytometry data were calculated with Statistica 12.0 software (Statsoft, Poland) and values at *p* < 0.05 were deemed significant. As data were not normally distributed, Mann-Whitney U test and Spearman’s rank correlation were used. Differences in gene expression calculated in Cuffdiff software were considered statistically significant when uncorrected p-value and FDR-adjusted q-value were both < 0.05. The Core Analysis in Ingenuity Pathway Analysis allowed to identify causes and effects related to differentially expressed genes in our dataset. Visualization of RNA-seq data as heat map was performed with ClustVis tool freely available at http://biit.cs.ut.ee/clustvis/#mathematics. Flow cytometry data were analyzed with BD FACS Diva v8.01 and FlowJo v7.6 softwares.

### Data Availability

Both next generation sequencing (NGS) raw and processed matrices are available in ArrayExpress database (http://www.ebi.ac.uk/arrayexpress) under accession number E-MTAB-5322.

## Electronic supplementary material

Supplementary Materials 1

Table S1

Supplementary Materials 2
