# Author Correction: Mild hypothermia provides Treg stability

**DOI:** 10.1038/s41598-017-17186-4

**Published:** 2017-12-15

**Authors:** Natalia Marek- Trzonkowska, Karolina Piekarska, Natalia Filipowicz, Arkadiusz Piotrowski, Magdalena Gucwa, Katrin Vogt, Birgit Sawitzki, Janusz Siebert, Piotr Trzonkowski

**Affiliations:** 1 Laboratory of Immunoregulation and Cellular Therapies, Department of Family Medicine, Medical University of Gdańsk, ul. Dębinki 2, 80-210 Gdańsk, Poland; 2 Department of Biology and Pharmaceutical Botany, Medical University of Gdańsk, al. Gen. J. Hallera 107, 80-416 Gdańsk, Poland; 3 Institute for Medical Immunology, Charité – Universitätsmedizin Berlin, corporate member of Freie Universität Berlin, Humboldt-Universität zu Berlin and Berlin Institute of Health, Augustenburgerplatz 1, 13353 Berlin, Germany; 4 Department of Family Medicine, Medical University of Gdańsk, ul. Dębinki 2, 80-210 Gdańsk, Poland; 5 Department of Clinical Immunology and Transplantology, Medical University of Gdańsk, ul. Dębinki 7, 80-210 Gdańsk, Poland

*Scientific Reports* **7**:11915; doi:10.1038/s41598-017-10151-1; Article published online 20 September 2017

The original version of this Article contained errors in the spelling of the authors Natalia Marek- Trzonkowska, Karolina Piekarska, Natalia Filipowicz, Arkadiusz Piotrowski, Magdalena Gucwa, Katrin Vogt, Birgit Sawitzki, Janusz Siebert & Piotr Trzonkowski which were incorrectly given as Marek- Trzonkowska Natalia, Piekarska Karolina, Filipowicz Natalia, Piotrowski Arkadiusz, Gucwa Magdalena, Vogt Katrin, Sawitzki Birgit, Siebert Janusz & Trzonkowski Piotr.

These errors have now been corrected in the PDF and HTML versions of the Article and in the accompanying Supplementary Material.

