# Supplementary material for: Mild hypothermia provides Treg stability
Source: Sci Rep. 2017 Sep 20;7:11915. doi: 10.1038/s41598-017-10151-1 (PMC5607276; doi:10.1038/s41598-017-10151-1)
Supplement: Supplementary file 1 — Supplementary Materials 1 [file 41598_2017_10151_MOESM1_ESM.doc]

**Mild hypothermia provides Treg stability**

Natalia Marek- Trzonkowska, Karolina Piekarska, Natalia Filipowicz, Arkadiusz Piotrowski, Magdalena Gucwa, Katrin Vogt, Birgit Sawitzki, Janusz Siebert, Piotr Trzonkowski

**INVENTORY OF SUPPLEMENTARY DATASET**

Legend of Table S1

Figure S1

Figure S2

Figure S3

Figure S4

**Table S1. Differentially expressed genes in Tregs cultured at 33°C based on NGS results.** Changed gene expression in Tregs cultured for 14 days at 33°C in relation to Tregs expanded at 37°C. Data were calculated with Cuffdiff as implemented in Cufflinks package (Trapnell et al. 2013) for three independent experiments for each culture condition. Genes with statistically significant change of expression (p- and q-values < 0.05) in Tregs expanded at 33°C as compared with those at 37°C were blue shaded, FPKM values for Tregs cultured at 37°C and 33°C are given as mean value for three replicates, log_2_ (fold_change) is the (base 2) log of the fold change y/x, p-value stands for uncorrected p-value of the test statistic and q-value for FDR-adjusted p-value of the test statistic. Gene nomenclature according to HUGO Gene Nomenclature Committee (HGNC) Database is used throughout the table.

**Figure S1**


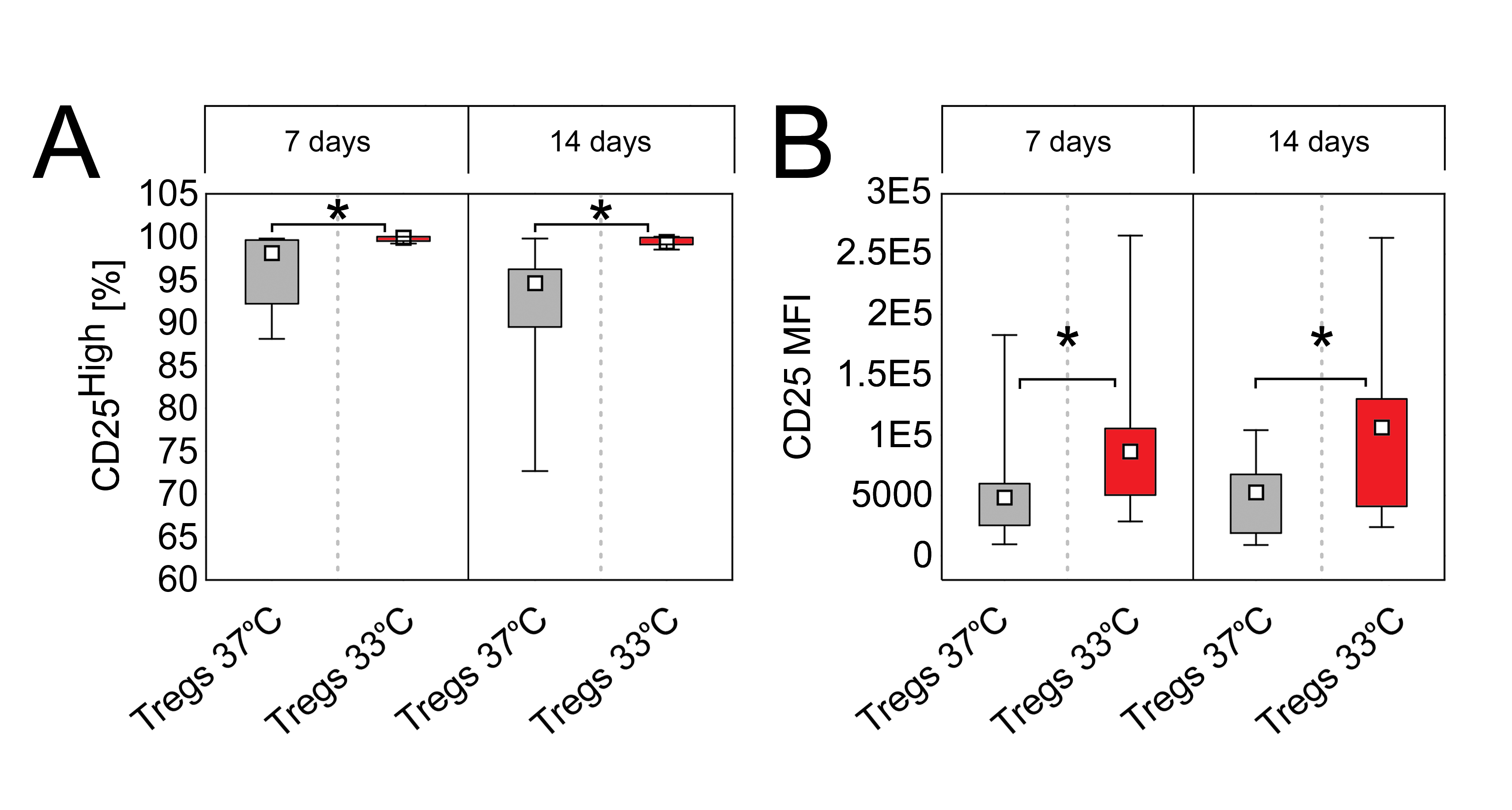


**Figure S1. Expression of CD25 in FoxP3^High^ Tregs expanded at 33 and 37°C.** (A) Frequency of CD25^High^ cells within CD4^+^FoxP3^High^ population on day 7 and 14 of Treg expansion at 37 and 33°C (gray and red symbols, respectively; n=11). (B) Intensity of CD25 expression (MFI) by CD4^+^FoxP3^High^CD25^High^ cells after 7 and 14-day expansion at 37 and 33°C (gray and red symbols, respectively; n=11). MFI is presented as value to power of 5 (e.g. 1E5=1x10^5^). The differences were calculated with Mann- Whitney U test and are presented as medians (min.-max.). **p*<0.05.


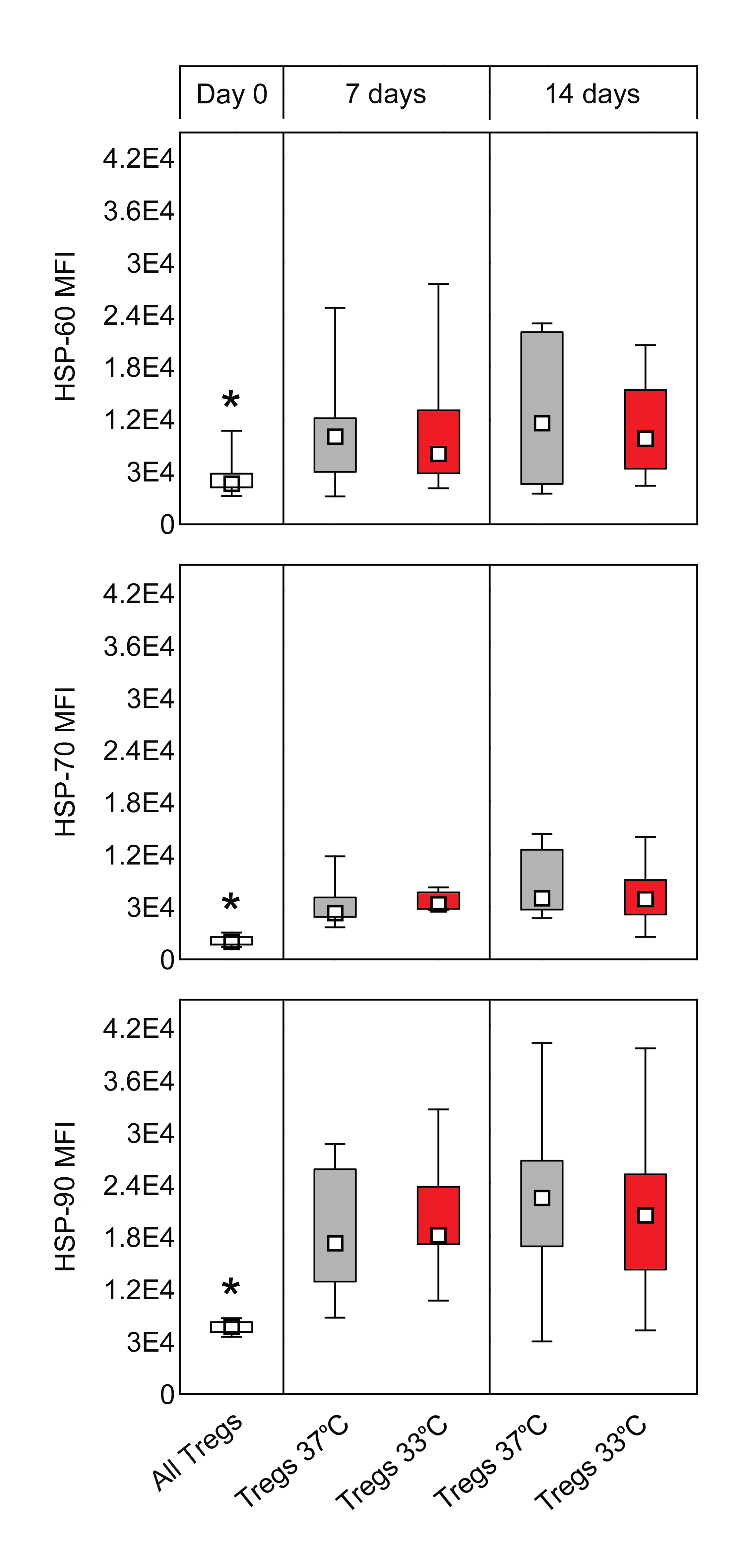
**Figure S2**

**Figure S2. Expression of HSP-60, HSP-70 and HSP-90 in Tregs expanded at 33 and 37°C.** Intensity of HSP-60, -70 and -90 expression (MFI) by CD4^+^FoxP3^+^ cells at day 0 and after 7 and 14-day expansion at 37 and 33°C (gray and red symbols, respectively; n=11). MFI is presented as value to power of 4 (e.g. 3E4=3x10^4^). The differences were calculated with Mann- Whitney U test and are presented as medians (min.-max.). **p*<0.05. On each chart, the asterisk indicates statistically significant difference between initial HSP expression in Tregs on day 0 and in Tregs expanded at 33 and 37°C on day 7 and 14.

**Figure S3**

**
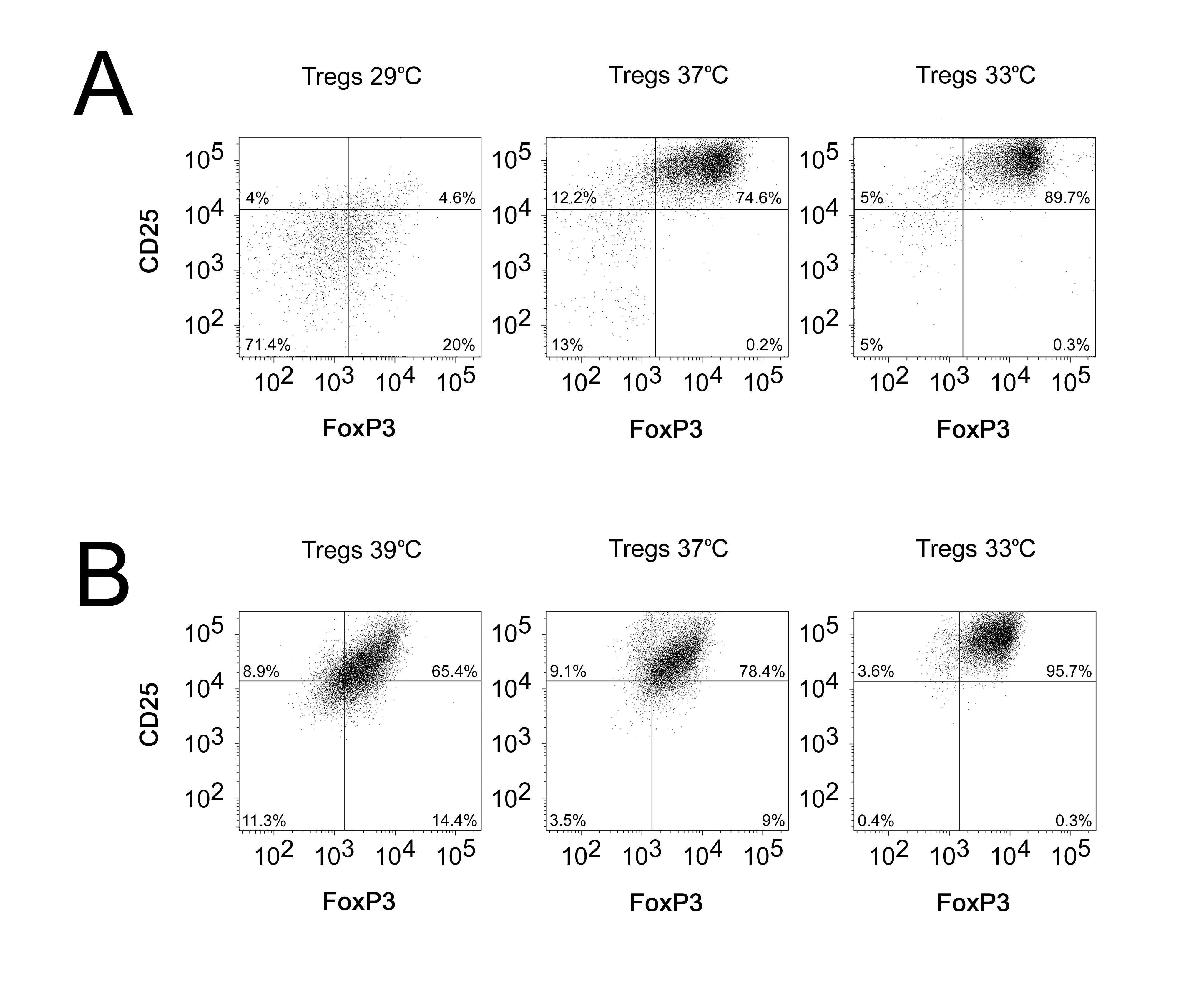
**

**Figure S3. Influence of temperature of 29°C and 39°C on expression of FoxP3 and CD25 in Tregs.** (A) Dot plots from one control experiment depicting FoxP3 and CD25 expression in Tregs cultured for 14 days at 29, 37 and 33°C. (B) Dot plots from one control experiment depicting FoxP3 and CD25 expression in Tregs cultured for 14 days at 39, 37 and 33°C. Frequency of CD4^+^ cells expressing CD25 and/or FoxP3 is shown for each temperature condition.

**Figure S4**

**
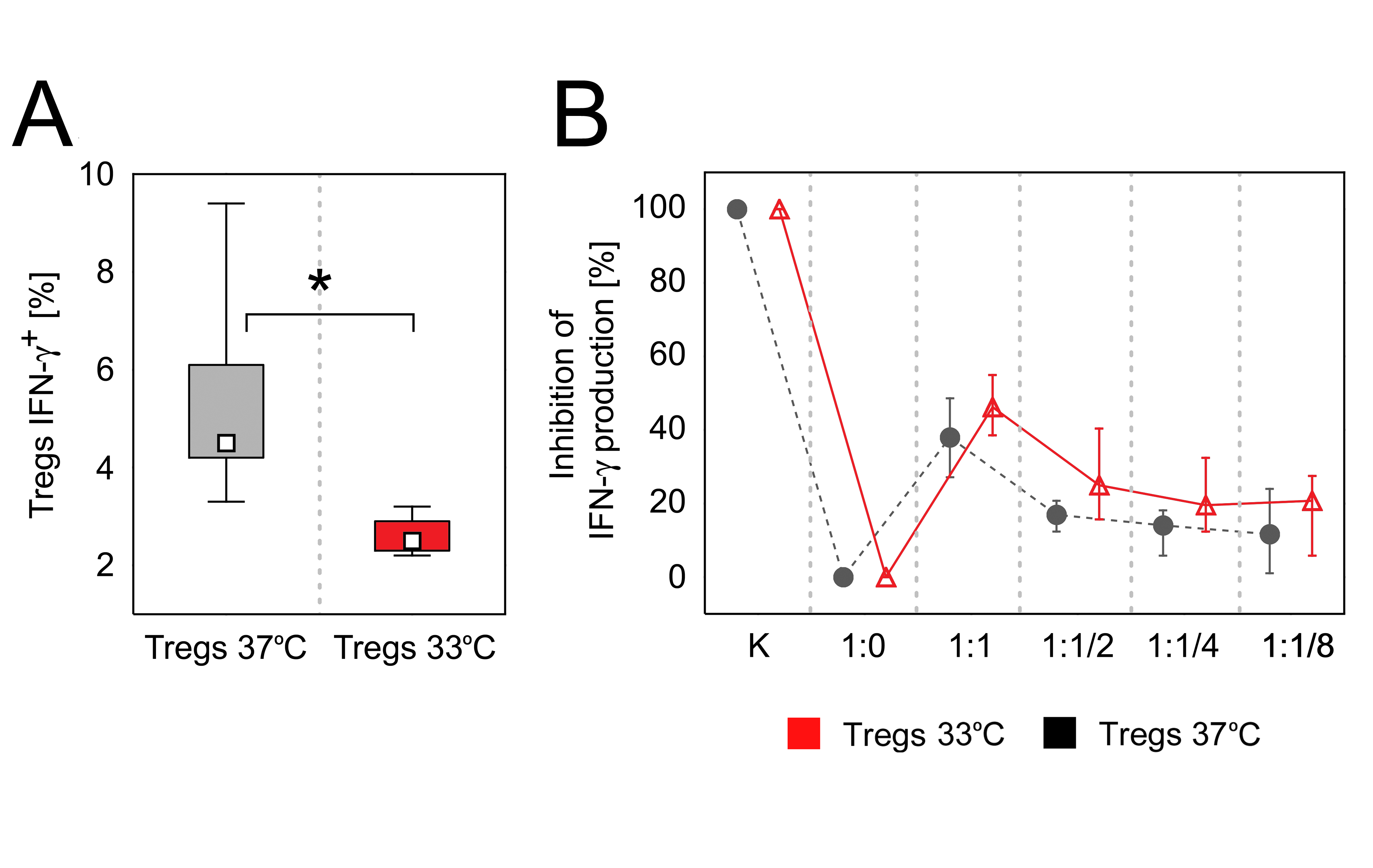
**

**Figure S4. Inhibition of IFN-γ production by Tregs expanded at 33 and 37°C.** (A) Frequency of IFN-γ^+^ cells on day 14 of Treg expansion at 37 and 33°C (gray and red symbols, respectively; n=5). (B)% of inhibition of IFN-γ** **synthesis in Teffs by Tregs expanded at 37 and 33°C (gray and red symbols, respectively; n=4). Results** **for various Teff:Treg ratios are shown. C1= control unstimulated Teffs cultured without Tregs, value corresponds to complete inhibition of IFN-γ production (100%). C2= control stimulated Teffs cultured without Tregs, value corresponds to complete lack (0%) of inhibition of IFN-γ production.
