# Supplementary material for: Mild hypothermia provides Treg stability
Source: Sci Rep. 2017 Sep 20;7:11915. doi: 10.1038/s41598-017-10151-1 (PMC5607276; doi:10.1038/s41598-017-10151-1)
Supplement: Supplementary file 3 — Supplementary Materials 2 [file 41598_2017_10151_MOESM3_ESM.pdf]

# Mild hypothermia provides Treg stability

Natalia Marek- Trzonkowska, Karolina Piekarska, Natalia Filipowicz, Arkadiusz Piotrowski, Magdalena Gucwa, Katrin Vogt, Birgit Sawitzki, Janusz Siebert, Piotr Trzonkowski

## INVENTORY OF SUPPLEMENTARY DATASET

Figure S5

**Figure S5. Top regulator effect network based on differentially expressed genes in Tregs expanded at 33°C.** The network was generated in Ingenuity Pathway Analysis and yielded the highest consistency score of 51.5. The middle row consists of molecules that are connected with upstream regulators (transcription regulators: IRF8, KLF2, CDKN2A, GFI1, NFKB1, BCL6 and RELA, presented in the top row) and downstream diseases and functions (bottom row).

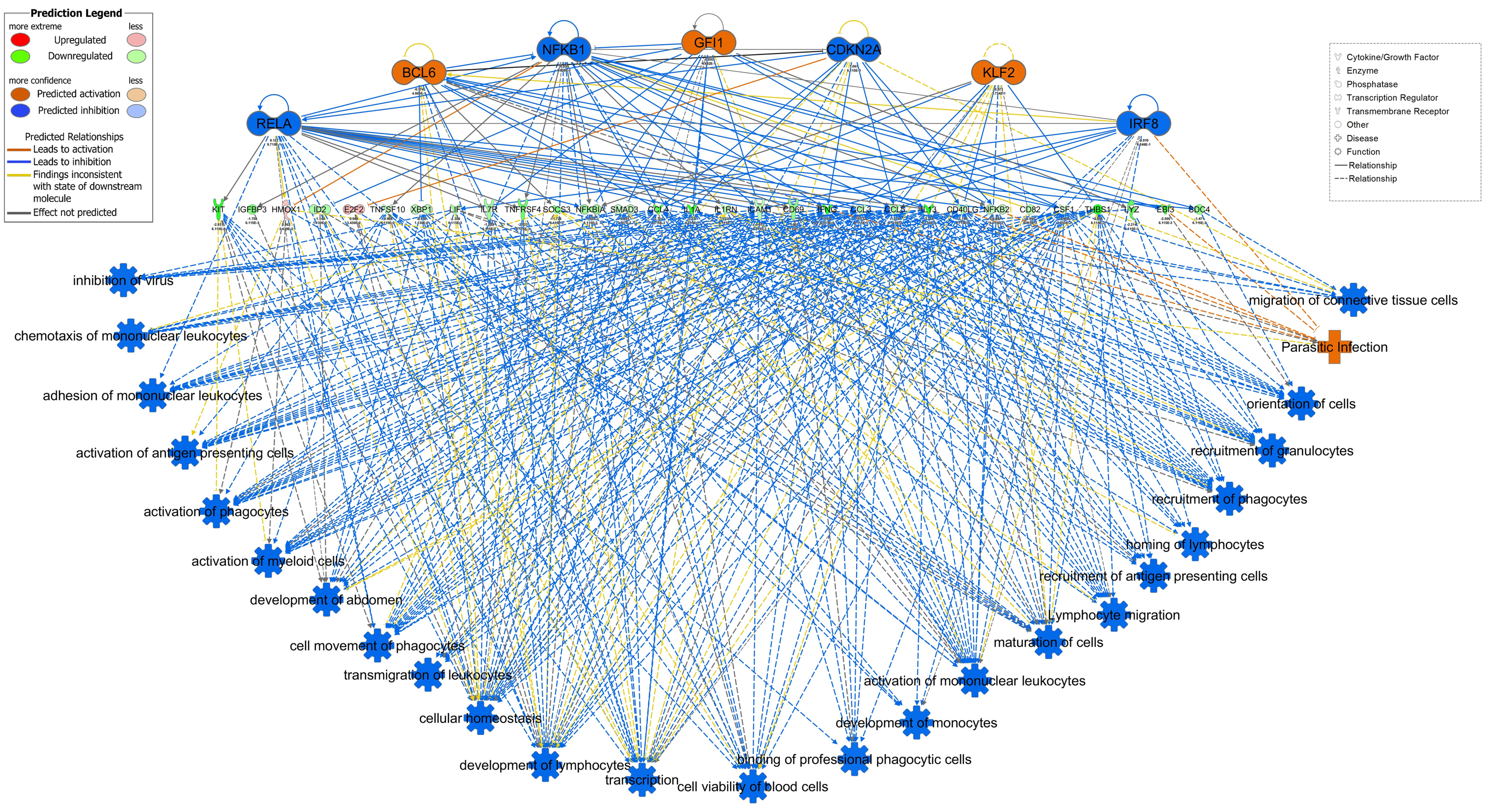


© 2000-2016 QIAGEN. All rights reserved.
